# Modern maize varieties going local in the semi-arid zone in Tanzania

**DOI:** 10.1186/1471-2148-14-1

**Published:** 2014-01-02

**Authors:** Ola T Westengen, Kristoffer H Ring, Paul R Berg, Anne K Brysting

**Affiliations:** 1 Centre for Development and the Environment (SUM), University of Oslo, Box 1166 Blindern, NO-0317 Oslo, Norway; 2 Centre for Ecological and Evolutionary Synthesis (CEES), Department of Biosciences, University of Oslo, Box 1066 Blindern, NO-0316 Oslo, Norway

**Keywords:** Crop evolution, Open pollinated varieties, Seed systems, Adaptation, Hybridization, Creolization

## Abstract

**Background:**

Maize is the most produced crop in Sub-Saharan Africa, but yields are low and climate change is projected to further constrain smallholder production. The current efforts to breed and disseminate new high yielding and climate ready maize varieties are implemented through the formal seed system; the chain of public and private sector activities and institutions that produce and release certified seeds. These efforts are taking place in contexts currently dominated by informal seed systems; local and informal seed management and exchange channels with a long history of adapting crops to local conditions. We here present a case study of the genetic effects of both formal and informal seed management from the semi-arid zone in Tanzania.

**Results:**

Two open pollinated varieties (OPVs), Staha and TMV1, first released by the formal seed system in the 1980s are cultivated on two-thirds of the maize fields among the surveyed households. Farmer-recycling of improved varieties and seed selection are common on-farm seed management practices. Drought tolerance and high yield are the most important characteristics reported as reason for cultivating the current varieties as well as the most important criteria for farmers’ seed selection. Bayesian cluster analysis, PCA and F_ST_ analyses based on 131 SNPs clearly distinguish between the two OPVs, and despite considerable heterogeneity between and within seed lots, there is insignificant differentiation between breeder’s seeds and commercial seeds in both OPVs. Genetic separation increases as the formal system varieties enter the informal system and both hybridization with unrelated varieties and directional selection probably play a role in the differentiation. Using a Bayesian association approach we identify three loci putatively under selection in the informal seed system.

**Conclusions:**

Our results suggest that the formal seed system in the study area distributes seed lots that are true to type. We suggest that hybridization and directional selection differentiate farmer recycled seed lots from the original varieties and potentially lead to beneficial creolization. Access to drought tolerant OPVs in combination with farmer seed selection is likely to enhance seed system security and farmers’ adaptive capacity in the face of climate change.

## Background

The current food price crisis has brought agriculture and food security back on top of the world’s development agenda [1]. In Sub-Saharan Africa (SSA) nearly a third of the population lives in chronic hunger and increased agricultural production is necessary to improve food security in the region [2]. This is why development agencies, governments and scientists have engaged in efforts to create a “Green Revolution for Africa” [3,4]. The goal is to reproduce the success of the green revolution that modernized agriculture in Asia and Latin America in the 1960s-1980s [5,6], but allegedly bypassed SSA [7]. The key technology factor in the green revolution was the development and dissemination of high yielding crop varieties [6,8]. Maize (*Zea mays* L.) is the main food crop for more than 300 million people in SSA and maize research and development (R&D) play a central part in the efforts to initiate a green revolution in the region [9]. Consequently, the tropical maize genome is under strong selection for increased yield and adaptation to African agroecologies [10,11]. Given the current challenge of drought and the projected negative impacts of climate change in SSA, drought tolerance is among the most important traits targeted in the new varieties [12,13]. A range of improved varieties spanning from open pollinated varieties (OPVs) and hybrids bred with conventional breeding techniques [14,15], as well as transgenic varieties [16] are currently being developed and promoted in SSA. Because climate change is an increasing challenge, development of improved climate-ready varieties is considered key to ensure food security in the years to come [13].

In order to enhance the delivery of the new varieties to farmers there is also a renewed focus on strengthening the African seed sector [17]. This entails strengthening links in the chain of public and private sector activities and institutions that produce and release certified seeds of officially registered varieties. This chain of activities and institutions is commonly referred to as “the formal seed system” [18,19]. Despite decades of attempts to formalize the African seed sector, the seed management and exchange taking place outside the formal institutions of variety development and distribution, i.e. “the informal seed system”, supplies a far larger share of the seeds than the formal system in SSA [20]. Three major aspects of importance to programs aiming at enhancing food security through seed system interventions are summarized in the concept “seed system security”: Seeds must be available, farmers must to be able to access them, and the seeds must be of a satisfactory quality [18].

A recent review concluded that the formal seed systems have been inefficient in delivering on the potential to create a maize based green revolution for Africa during the past two decades [9]. There are challenges connected with all three aspects of seed system security in the maize seed systems in SSA, including problems with the quality of the seeds available. In addition to seed health and germination rate, the genetic quality, which refers to both adaptability and purity of the varieties, is an important element of seed quality [18,19]. Maize is a diverse crop whose adaptations are highly location specific and the formal seed system has not been able to deliver sufficient locally adapted varieties to the heterogeneous agroecologies in the region [9]. There is also concern about the ability of some of the formal maize systems of SSA to deliver seeds with acceptable varietal purity [17,21]. Studies of genetic integrity of seed lots have documented genetic differentiation, genetic contamination and mislabeling of improved varieties [21,22]. The persistent challenges in achieving seed and food security in developing countries have led some authors to suggest that seed system interventions should shift focus from approaches aiming at replacing the informal system with a formal system into approaches that build on the strengths of the existing informal seed systems [19,23].

Informal seed systems include saving from own harvest, farmer-to-farmer seed exchange, and purchase from local markets [24]. Not only traditional varieties are sourced through such informal channels, but also farmer recycled improved varieties. In a cross-pollinating crop like maize, recycling of improved varieties can lead to potentially beneficial hybridization with local varieties grown in proximity, a phenomenon known as “creolization” [25]. Extensive farm saving and recycling of improved varieties is a concern to those who see a well-functioning supply of certified seeds as a prerequisite for long term sustained growth in maize production in SSA [11]. However, studies by Bellon et al. [25,26] of creolization in the center of diversity of maize in Mexico present another perspective and suggest that farmers expand on the benefits of improved varieties through purposeful adaptation to local agronomic conditions and consumption preferences. To our knowledge creolization has not been studied in SSA, despite the fact that the necessary conditions (i.e. farmer-recycling, hybridization and on-farm selection) are documented in other studies from the region [27-29]. Although several authors have stated the interesting prospect of investigating creolization of improved varieties with molecular methods [10,26], the phenomenon has as far as we know only been explored in the socio-economic development literature.

We here present a case study of formal and informal elements of a local seed system in the semi-arid agroecological zone in the district Morogoro in Tanzania. Agriculture is the most important activity in people’s livelihoods and maize is the major staple. The local agroecology is already marginal for maize production and future maize production is projected to be severely negatively affected by climate change unless adaptation measures are taken. In another paper on adaptation to climate stress in the area, we show that households cultivate both improved and local varieties and that seeds originally sourced from the formal system have entered the informal system (Westengen & Brysting, in revision). In this paper we explore the consequences of seed management in-depth by studying genetic diversity and differentiation in the local maize seed system. We address the following research questions: 1) Are improved varieties distributed through the formal seed system true to type (genetically identical) as they pass through the formal seed chain from breeder’s seeds to certified seeds? 2) What is the genetic effect of on-farm seed management when the improved varieties enter the informal seed system? In order to address the first question we assess the genetic structure of seed lots of the most common varieties through the different stages of seed production in the formal seed system from breeder’s seeds, through basic seeds to commercial seeds. In order to address the second question we genotype seed lots of the same varieties after entering the informal seed system through farmer-recycling. We define a seed lot as all seeds of a named OPV from the same source population, be it from a field managed by a field manager in the formal system or from a farmer’s field in the informal system. We use single nucleotide polymorphisms (SNPs) to genotype seed lots and include both randomly selected markers and a set of candidate markers for directional selection.

## Methods

### Characterizing the local seed system

We used both qualitative and quantitative research methods to characterize the local seed system. Qualitative data on the formal system was obtained through interviews of players in the formal seed system in the region: Scientists at the Sokoine University of Agriculture in Morogoro, breeders in the public sector, government officials in the public institutions involved in seed testing and certification, local extension workers, and representatives of major seed companies in the region.

The village Mangae in the Morogoro district in Tanzania was selected for a survey of variety use and seed management. The village was surveyed as part of a study in the semi-arid agroecological zone including also another village in Dodoma district (Westengen & Brysting, in revision) and the data presented here is from the same survey. We selected this village because 1) maize plays a predominant role in the agricultural system and 2) small-holder livelihoods in the village are typical for those targeted in the new maize seed system interventions. A total of 159 randomly selected households were surveyed using a structured questionnaire, including questions about crop variety use and seed management practices.

Based on the survey results we selected the two most common improved maize varieties, Staha and TMV1, for genetic analysis. We sampled seed lots of the two OPVs at different stages in the seed system; breeder’s seeds, basic seeds, certified commercial seeds and different generations of the OPVs sampled from farmers’ fields in Mangae. Three of the seed lots sampled on-farm (*Staha local 1, Staha local 2* and *TMV1 local*) were reportedly used for one generation (i.e. we sampled seeds from the first harvest) while *Staha recycled* was reportedly recycled for 10 generations. In addition we sampled seed lots of two local varieties common in the village. A research permit for the fieldwork in Tanzania was obtained through Sokoine University of Agriculture. Collecting of seeds from farmer’s fields was done under prior informed consent with farmers and village authorities. The seeds sampled on-farm were randomly drawn from different ears in farmers’ storage. Breeder’s seeds were provided by the primary breeder of Staha and TMV1, Alfred Moshi.

### Genotyping and molecular data analysis

We selected SNPs to include in this study on the basis of a genetic structure and adaptation analysis of a panel of African maize [30] done with the MaizeSNP50 array [31]. Out of 43,963 SNPs, we included a total of 144 SNPs belonging to three groups: 1) Nine candidate SNPs earlier suggested to be positively associated with maximum temperature during the growing season in the African panel based on a regression model [30]; 2) Thirty-five SNPs suggested to be under positive selection in the African panel based on F_ST_ values using the program LOSITAN [32] and; 3) One hundred randomly selected SNPs evenly distributed on all 10 maize chromosomes (Additional file 1: Table S1). A total of 109 of the SNPs included are located in known or putative genes identified with the Panzea marker search database (http://www.panzea.org) and the maize sequence on the Gramene database (gramene.org).

Germination of the sampled seeds and DNA extraction were done as described in [30]. A total of four multiplexes, consisting of 144 SNPs, were designed using the software SpectroDESIGNER™ v3.0 (Sequenom Inc, San Diego, CA). Genotyping was done with the Sequenom MassARRAY iPLEX platform [33] according to the manufacturer's protocols (Sequenom), using the Sequenom MassARRAY Analyzer 4 instrument and the MassARRAY Workstation v3.3 software (Sequenom). Manual inspection of all results was carried out using the MassARRAY Typer software version 4.0 (Sequenom).

We measured the global and per SNP observed heterozygosity (H_O_) and expected heterozygosity (H_E_) according to Nei (1987) [34] within each of the 12 sampled populations and the pairwise genetic differentiation between populations applying the F_ST_ estimator by Weir and Cockerham (1984) [35] using the program ARLEQUIN 3.5 [36] with 1000 permutations to determine statistical significance. Population structure and individual genome admixture were estimated with the Bayesian Markov Chain Monte Carlo (MCMC) clustering model implemented in the program STRUCTURE v.2.3.3 [37,38]. We used the correlated allele frequency and admixture model with no prior population information to estimate the posterior probability of membership of individual genomes from *K* populations. We ran 10 independent runs for each value of *K* from 2 to 18 with a burn-in period of 2 × 10^5^ followed by 2 × 10^5^ iterations. The most probable number of groups, *K*, was determined with STRUCTURE HARVESTER [39], calculating the mean Ln likelihood and variance per *K* value and the *ad-hoc* measure in change in likelihood between successive *K* values, delta *K *[40]. The results were visualized with the program DISTRUCT [41], using the runs with the highest Ln likelihood for the selected values of *K*.

We performed principal component analysis (PCA) using the R (http://www.r-project.org) package ADEGENET [42] combining SNP data on the formal sector Staha and TMV1 samples with data on an African maize panel [30]. The PCA method is free of assumptions of Hardy-Weinberg equilibrium or absence of linkage disequilibrium and is a good alternative and supplement to the STRUCTURE algorithm [43]. Furthermore, we simulated a hybrid population between the formal seed system populations of Staha and the local varieties and plotted the two first components using the *hybridize* function and the PCA function in ADEGENET.

We performed a scan for evidence of directional selection across seed system stages using the Bayesian method implemented in BAYENV [44]. We used stages in the local seed system of Staha as an ordinal environmental variable in a similar manner as van Heerwarden et al. (2012) [45] used breeding era. We defined three stages: 1) the formal stage (including *Staha breeder’s seeds*, *basic seeds* and *commercial seeds*); 2) used 1 year (including *Staha local 1* and *Staha local 2*) and; 3) recycled 10 yrs (*Staha recycled*). A total of 116 SNPs were polymorphic between the Staha populations; 82 random SNPs and 34 candidates for selection. As a basis for the null model, we excluded the candidate SNPs and used the random SNPs to estimate 10 covariance matrices in BAYENV using MCMC iterations ranging from 5000 to 50,000. We tested each SNP in the dataset for correlation with the stage variable and calculated Bayes factors. The consistency of the results was checked with multiple runs of BAYENV using different iterations (30,000 and 50,000) and random seeds for the MCMC algorithm.

## Results

### Varieties and seed management

Maize is the most important crop in Mangae, both in terms of number of households cultivating it and in terms of total land acreage allocated. Ninety-eight percent of the households in Mangae cultivate maize to some extent. The median area cultivated to maize is 4 acres (average 4.5 acres). The number of households cultivating the different varieties and the reasons they report for growing them are listed in Table 1. Households report that in 22% of the fields cultivated with improved varieties, the seeds have been recycled on-farm for at least two years. However, it is difficult to determine the share and extent of farmer-recycled seeds since a considerable share of the improved variety seeds in the study area are sourced through the informal seed system and the seeds might already be recycled before they are first sourced by the households surveyed.

**Table 1:** Maize varieties cultivated in Mangae, Morogoro, and the households’ reason for growing them

**Variety**	**Maize fields planted (N = 222)**^**2**^	**Drought tolerance**	**Yield**	**Tastiness**	**Biotic stress resistance**
**Katumani**	6	na	na	na	na
**Kito**	27	0.41	0.59	0.07	0.00
**Staha**	82	0.47	0.65	0.06	0.01
**TMV 1**	69	0.25	0.53	0.01	0.01
**TAN250**	1	na	na	na	
**Local**	19	0.58	0.32	0.15	0
**Other**^**1**^	18	na	na	na	na

The frequency of the respondents’ reason for growing a given variety is based on the average for the binary variable: 1, if this is a reason for growing the variety and 0, if it is not.

^1^Undetermined origin.

^2^Numbers add up to more than the number of households (156) because some households cultivate different varieties in different fields.

na = data not available because of too few observations.

Information about the improved varieties cultivated in the study village is listed in Additional file 2: Table S2. The two varieties Staha and TMV1 are cultivated on about two-thirds of the maize fields in this survey (Table 1). Both varieties are developed under the National Maize Research Program (NMRP), at the Agricultural Research Institute (ARI) Ilonga in Morogoro [46]. The breeders’ seeds are supplied by ARI to the seed production farms of the Agricultural Seed Agency (ASA), a semi-autonomous body under the Ministry of Agriculture, Food Security and Cooperatives which has the mandate to produce, process and market sufficient seeds of public varieties (http://www.asa.or.tz/asaa/). ASA produces certified seeds through its own seed farms, as well as contract growers applying the FAO Quality Declared Seed (QDS) scheme with small scale farmers located in various parts of the country [47]. Some of the seeds are distributed by private seed companies, while ASA also distributes and markets their own seed. Seed quality control is the responsibility of the Tanzania Official Seed Certification Institute (TOSCI).

Out of the 156 maize cultivating households in Mangae, 113 (72%) report that they normally select seeds for next year’s planting (Table 2). Seed selection is most frequently done as selection of cobs during harvest and the most important plant trait for seed selection is drought tolerance (31%) while the most important cob trait is seed filling (44%).

**Table 2:** On-farm seed management practices reported by households (HHs) in Mangae, Morogoro

**Practice**	**Trait**	**Share of HHs**
Seed selection		72%
	Plant drought tolerant	31%
	Plant resistant to lodging	13%
	Plant true to type	10%
	Plant flowering time	3%
	Cob well filled	44%
	Cob of large size	22%
Spacing between plots with different varieties		43%
Staggered planting of different varieties		29%

The last column refers to the share of households conducting the practice and selecting seeds on the basis of the listed traits.

### Genetic diversity and population structure

Out of the 144 designed SNPs, 131 were polymorphic and showed good clustering/separation in this material and were hence included in the subsequent analyses (Additional file 1: Table S1). The final dataset includes 80 individuals from 12 sampled seed lots (Table 3). The number of polymorphic loci within samples range from 100 in *Staha commercial* to 47 in *Local variety 2*. The within samples observed heterozygosity range from 0.298 in *Staha local 1* and *Staha breeder’s seeds* to 0.436 in *Staha recycled*. The overall genetic differentiation is F_ST_ = 0.18 and pairwise F_ST_ values (Additional file 3: Table S3) range from the insignificant values 0.01 and 0.04 between breeder’s seeds and commercial seeds in Staha and TMV1, respectively, to 0.38 and 0.35 between basic seeds of the two OPVs and *Local variety 2*. The F_ST_ value between breeder’s seeds of the two OPVs is 0.14.

**Table 3:** Maize populations in Mangae, Morogoro, surveyed by SNP analysis

**Seed lot name**	**Source**	**Characteristic/color**	**Number of plants (in final analysis)**	**Number of polymorphic loci**	**H**_**O**_	**H**_**E**_
Staha breeder’s seed	ARI Ilonga	White Flint-Dent	8(8)	95	0.298	0.371
Staha basic seed*	ASA seed farm	White Flint-Dent	8(8)	79	0.314	0.344
Staha commercial	Seed shop	White Flint-Dent	8(7)	100	0.309	0.383
Staha local 1	Farmer in Mangae	White Flint-Dent	8(7)	99	0.298	0.343
Staha local 2	Farmer in Mangae	White Flint-Dent	8(7)	80	0.365	0.373
Staha recycled	Farmer in Mangae	White-Purple	8(8)	77	0.436	0.396
TMV1 breeder’s seed	ARI Ilonga	White Flint	8(7)	88	0.307	0.377
TMV1 basic seed	ASA seed farm	White Flint	8(5)	74	0.401	0.402
TMV1 commercial	Seed shop	White Flint	8(3)	66	0.318	0.489
TMV1 local	Farmer in Mangae	White Flint	8(8)	77	0.339	0.378
Local variety 1	Farmer in Mangae	Red	8(6)	88	0.344	0.371
Local variety 2	Farmer in Mangae	Brown-White	8(6)	47	0.317	0.411

Observed heterozygosity (H_O_) and expected heterozygosity (gene diversity) (H_E_) calculated according to Nei (1987) [34].

*The term “Basic” used by the Organization for Economic Cooperation (OECD) is here used instead of the term “Foundation” seeds from the Association of Official Seed Certifying Agency (AOSCA), but the terms are used interchangeably in Tanzania.

**Figure 1:**
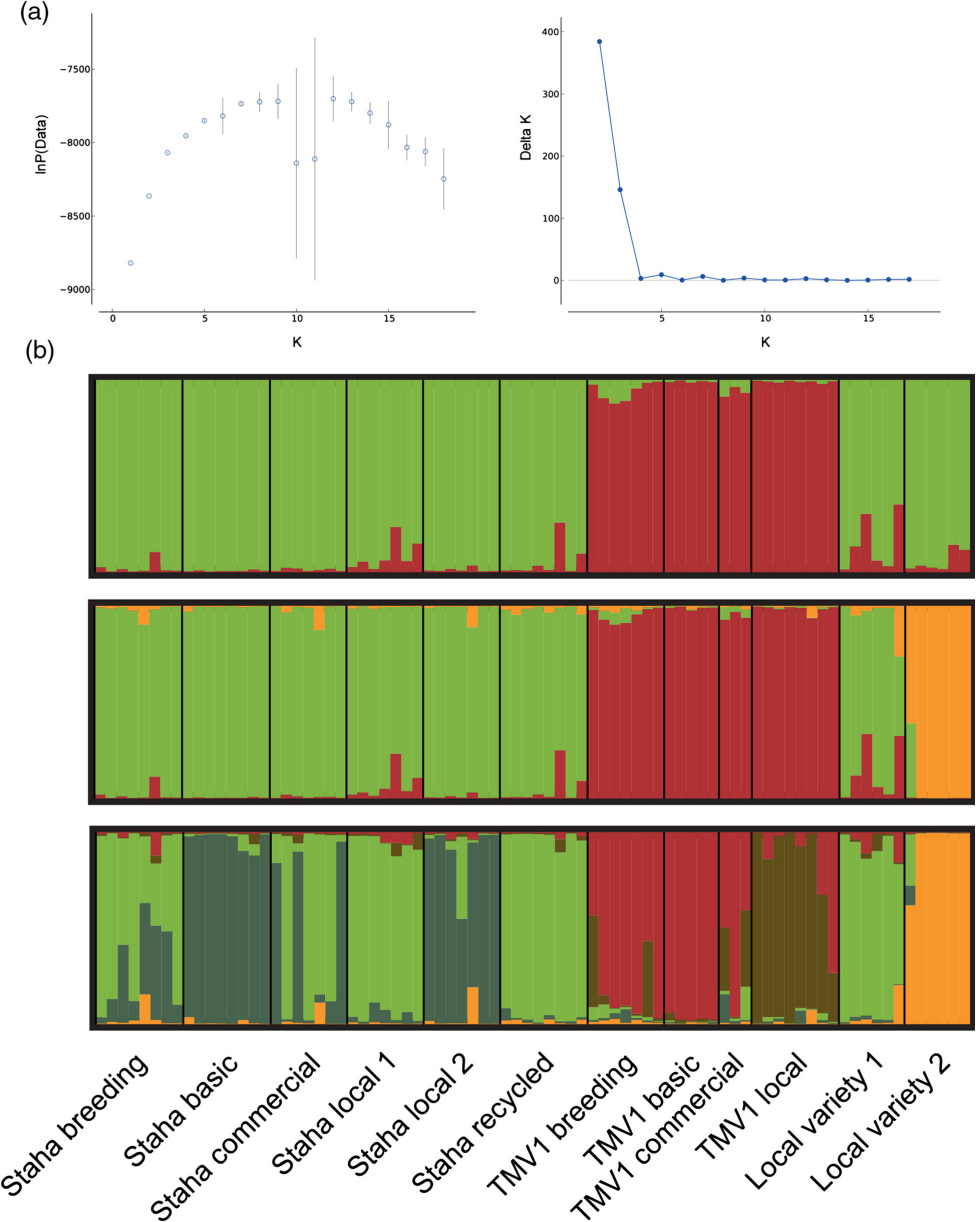
**Population structure in the OPV Staha and local varieties of maize in Mangae, Morogoro. (a)** Left hand side: Mean Ln probability of the data for 10 runs for each value of *K* from 1 to 18, with standard deviations. Right hand side: Plot of Evanno’s delta *K* for each value of *K***(b)** Structure plots of the assignment probabilities in different samples of OPVs. Each sample is represented by a bar. The plots are based on the highest probability run for *K* = 2, 3 and 5, respectively (from the top to the bottom).

Bayesian cluster analysis of Staha and the two local varieties shows that the likelihood increases when assuming five populations (*K* = 5), with an increase in variance thereafter. There is also a small increase in delta *K* for *K* = 5, but the value is much lower than for *K* = 2 and *K* = 3 (Figure 1a). Assuming two or three populations clearly distinguishes between Staha and TMV1, with all Staha samples clustering together with both local varieties at *K* = 2 and one of the local varieties forming its own cluster at *K* = 3. When assuming five populations, a subdivision within both Staha and TMV1is detected. The population subdivision in Staha is visible in all seed lots from the formal system with several admixed individuals both in *Staha breeder’s seeds* and *Staha commercial* (Figure 1b). Applying a >80% threshold to define membership to one of the two model-based populations, all individuals in the *Staha local 2* seed lot are clearly assigned to the same population as the individuals in the *Staha basic* seed lot, and all individuals in *Staha local 1* and *Staha recycled* are assigned to the other population. *TMV1 local*, the only on-farm collected population of TMV1*,* clusters separately from most individuals in the formal system samples, but the population, to which it is assigned, is also present in admixed individuals in the *TMV1 breeder’s seeds* and *TMV1 commercial* samples.

**Figure 2:**
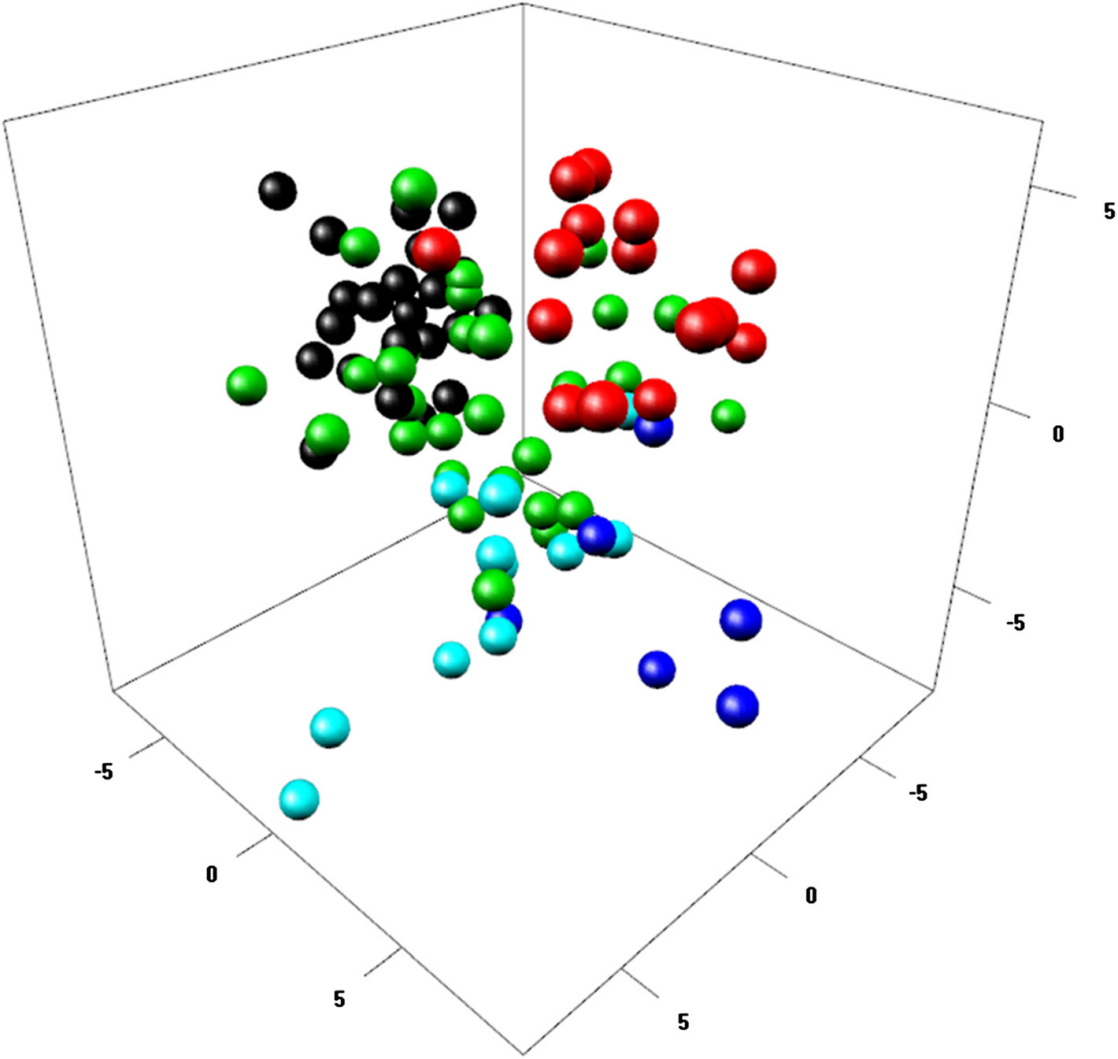
**Genetic structure of the OPVs TMV1 and Staha in context of an African maize panel.** PCA plot (three first components) of Staha and TMV1 in the context of a panel of African maize: Staha (black); TMV1 (red); East Africa (green); West Africa (blue); Sahel (turquoise).

**Figure 3:**
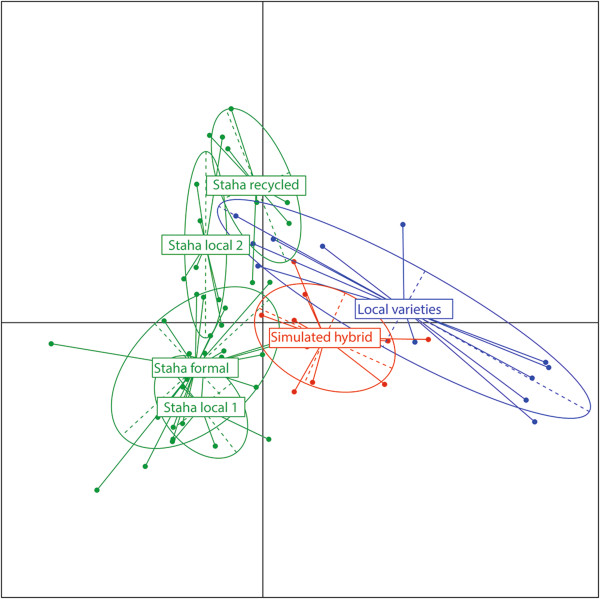
**Simulation of hybridization between the OPV Staha and local varieties.** PCA plot (two first components) of samples of Staha from the formal seed system and three different populations sampled on-farm: *Staha local 1*, *Staha local 2* and *Staha recycled* (all green), a sample of local varieties (blue) and a simulated hybrid between Staha from the formal system and the local varieties (red).

Multivariate analysis confirms the clear distinction between Staha and TMV1 in Mangae. The PCA plot of Staha and TMV1 in the context of the African panel in [30] shows the affinity of the two OPVs with the East African cluster (Figure 2). Visualization of the two first components of the PCA, where the three formal system Staha seed lots (*breeder’s seeds*, *basic seeds* and *commercial*) are merged and the two local varieties are merged, shows that two of the on-farm collected Staha populations (*Staha local 1* and *Staha local 2*) overlap with the formal system Staha while *Staha recycled* clusters separately and partly overlaps with the local variety cluster (Figure 3). *Staha recycled* seems, thus, more differentiated from the formal system group than the two Staha populations that had been grown on-farm for only one generation. The simulated hybrid between the formal seed system populations and the local varieties clusters intermediately and overlaps with both parent populations as expected.

**Figure 4:**
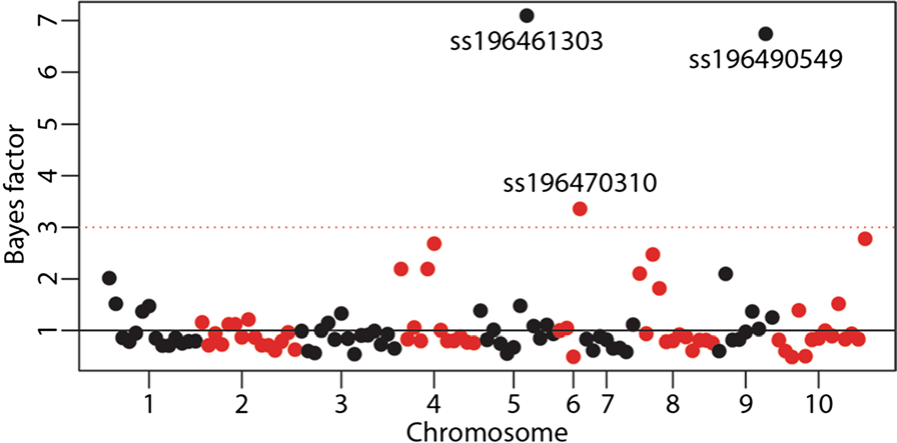
**Evidence for directional selection.** Manhattan plot of SNP association with seed system stage based on mean Bayes factor from 10 replicate runs of BAYENV [44]. The SNPs are plotted according to chromosome and position at chromosome along the Xaxis. Chromosomes 1–10 alter between black and red color. The red dotted line at 3 indicates a positive Bayes factor according to Kass and Raftery (1995) [48].

The scan for evidence of directional selection across seed system stages identifies three SNPs consistent between all 10 replicates. The Bayes factors for these SNPs vary between 3.5 and 7.1 which is “positive” according to the scale suggested by Kass and Raftery (1995) [48] (Figure 4). The three SNPs are all among the candidates for positive selection and two are located in known putative protein coding genes with known orthologous in rice and sorghum (GRMZM2G146041 and GRMZM2G028758).

## Discussion

### The formal seed system

Ideally, the formal seed system should provide improved and certified high yielding quality seeds that are well adapted to local agroecologies. However, various problems, ranging from seed production factors to policy issues, affect the effectiveness of the formal seed systems in producing and delivering seeds in SSA [9,17,20]. Compared to other crops maize has the highest adoption rate of improved varieties in SSA and Smale et al. (2013) [9] estimate that 44% of the maize area in Eastern and Southern Africa is planted to improved varieties. Within the region, Tanzania is at the lower end of the adoption statistics. Based on the sale of certified seeds, Langyintuo et al. (2010) [17] estimate that only about 18% of the maize area in Tanzania is planted to fresh improved maize seeds, while Smale et al. (2013) [9] suggest that the adoption figure in Tanzania can be adjusted to 22% if farm-saved seeds are factored in. In addition to the limited capacity to supply seeds, there are also problems with regard to the physical genetic quality of some of the seeds distributed by the formal system in SSA, i.e. that seed lots are not true to type [9,21,23,49]. Our study reveals that improved varieties are integrated in the local seed system in the study area. The majority of the households in the assessed community grow improved maize varieties while at the same time practicing recycling and seed selection. Our findings are only valid for the surveyed community, but they show that adoption statistics may leave a substantial share of the improved seeds undetected because there is a low seed replacement rate and the informal seed system supplies a large share of the seeds. OPVs distributed by the public R&D system in Tanzania dominate in the study area. These varieties were bred for the semi-arid conditions by breeders at the Ilonga ARI station in the same district and officially released in the 1980s. Breeder’s seeds of the two most prominent varieties, Staha and TMV1, are still maintained at the research station, and a Seed Farm managed by the public seed agency ASA produces basic seeds for further multiplication.

The two OPVs are clearly distinguished by classical F_ST_ measures, as well as by Bayesian cluster analysis and PCA. The genetic differentiation between the two OPVs (F_ST_ = 0.14) is in the higher end of the F_ST_ values obtained in an analysis of differentiation between heterotic groups of inbred lines from eastern and southern Africa [50] and similar to the mean F_ST_ between 10 OPVs from Zimbabwe [21]. Despite this differentiation, the PCA (Figure 2) shows that when considered in the context of a diverse panel of predominantly local maize varieties from across Africa, both Staha and TMV1 show affinity with East African varieties. This affinity can be explained by the presence of East African breeding material in the pedigree of both OPVs (Additional file 2: Table S2). Furthermore, the PCA reveals that Staha has stronger affinity with the rest of the East African varieties than does TMV1. The similarity with local varieties is also apparent from the STRUCTURE plot (Figure 1) where the seed lot of one of the local varieties is assigned to the same modeled ancestral population as Staha for all three assumed values of *K*.

OPVs that are not under strong selection pressure and not being mixed with unrelated varieties should have stable allele frequencies over generations. Deviations from this pattern can be used to test genetic purity in seed lots [21]. Assessing the genetic identity and purity of a diverse panel of tropical inbred lines, Semagn et al. (2012) [22] established a threshold of >5% allelic difference to determine if two lines were different from each other. Since OPVs are heterogenous populations, it is difficult to establish a similar threshold of genetic differentiation to determine whether or not the OPVs analyzed are true to type across the formal seed system stages from breeder’s seeds to marketed certified seeds. The F_ST_ values between the breeder’s seeds and basic seeds of both Staha and TMV1 (0.092 and 0.080, respectively) are higher or at the higher end of the values reported in Warburton et al. (2010) [20] between different bulks of the same varieties (0.000 to 0.084). Furthermore, compared to the values reported in the same study for seed lots purposefully “contaminated” with 5-50% seeds from unrelated populations, the difference between breeder’s and basic seeds in this study is also in the higher range, suggesting possible contamination in this critical stage of the seed multiplication. However, closer inspection of our results does not support the conclusion that the genetic purity of the OPVs is compromised in the formal seed system. First, the F_ST_ values between the breeder’s seeds and the commercial seeds are insignificant in both Staha and TMV1, thus the differentiation at the earliest stage of seed multiplication is no longer visible after the final production of certified seeds. Second, the Bayesian cluster analysis shows that individual seeds of both *Staha breeder’s seeds* and *Staha commercial seeds* are assigned to the same modeled ancestral population as *Staha basic seeds*. We therefore suggest that the substructure reflects the heterogeneity of the OPVs rather than genetic contamination or different origin of the basic seed lot. Thus, in the case of the two OPVs studied here we do not find evidence indicating that the formal seed system distributes seeds that are not true to type; the seeds sold in the seed shop are genetically similar to the breeder’s seeds maintained and distributed by the responsible research station.

The survey of farmers’ reason for growing the different varieties reveals that yield and drought tolerance are more important reasons than tastiness and resistance to biotic stress. Drought tolerance is more frequently reported as a reason for growing Staha than the other improved varieties in the area. Drought tolerance is also a characteristic of Staha reported by ASA, and the name actually means “resistant to drought” in Kiswahili. The drought tolerance in Staha is attributed to the share of Tuxpeño breeding material in its pedigree. The Tuxpeño breeding material was introduced by CIMMYT and originates from the Tuxpeño maize race from the Oaxaca-Chiapas lowland region in Mexico. It is highly productive and some of the elite maize populations derived from it are specifically bred for drought tolerance [10,51]. Tuxpeño elite populations have been widely used in maize breeding in the tropics and also the improved OPVs studied in Bellon and Taylor (1993) [52] were Tuxpeño derived. This study reported that farmers actually ranked the improved OPVs as being less drought tolerant than the local landraces. Similarly, in our study, farmers more frequently cite drought tolerance as a reason for growing local varieties than for any of the improved OPVs. Cairns et al. (2013) [53] recently reported that elite genetic resources bred for drought tolerance often fail to perform well under combined drought and heat stress. Genetic control of performance under abiotic stress is complex, involving different trait loci for heat and drought tolerance as well as for the two stresses combined [53]. Thus, improved varieties promoted as drought tolerant might fail when facing multiple abiotic stresses in farmers’ fields. However, the fact that Staha and TMV1 are still dominating in farmer’s fields nearly three decades after their first release suggests that they are well adapted to the local agricultural practices and a closer look at how these OPVs are integrated in the informal elements of the local seed systems offers interesting insights relevant for the new breeding and seed system initiatives.

### Farmer recycling and creolization

A rich body of literature on farmer management of maize varieties in the crop’s center of origin in present day Mexico has revealed that varieties are maintained as open genetic systems with frequent gene flow occurring between landraces and between landraces and improved varieties [54,55]. This dynamic view has implications for conservation as well as for efforts to introduce improved varieties. One important finding is that farmers seem to expand on the benefits of improved varieties through the process of creolization [25,26]. Creol varieties are hybrids between local varieties and improved varieties that may result from both intentional and unintentional crossing in farmers’ fields. Bellon et al. (2006) [25] found support for the hypothesis that farmers combine desirable traits from improved and traditional varieties through this process. Studies of creolization thus suggest that the genetic change following recycling and hybridization can be beneficial rather than detrimental, which is the common assumption in most adoption studies. In our survey of farmers’ seed management practices, we find that about half of the maize farmers in the study area are concerned with variety maintenance and keep varieties separate either in time, by staggered planting, or in space, by maintaining a distance between plots. However, we also find that more than 70% of the farmers practice selection and that for most of them the reason for doing this is to ensure that they have healthy seeds for next year’s planting.

Analyses of genetic structure show that seed lots of the OPVs sampled on-farm after one or more growing seasons are significantly differentiated from the seed lots sampled from the formal system. Within Staha seed lots, the pairwise F_ST_ value between the breeder’s seed and the one which has been recycled on-farm for 10 years, is highest (F_ST_ = 0.092) and within TMV1, the pairwise F_ST_ value between breeder’s seeds and the seed lot used one year is even higher (F_ST_ = 0.116). This level of differentiation is higher than what we find among seed lots from the formal system and both the Bayesian cluster analysis and the PCA indicate that genetic changes probably are due to other factors than mere population heterogeneity. A CIMMYT literature review of the phenomenon of farmer-recycling states that genetic differentiation from the original OPV of recycled seed lots can be due to genetic drift, mutation, segregation, hybridization or natural- and farmer-selection [27]. Distinguishing between these factors is difficult, but a closer look at Staha suggests that both hybridization and selection probably play a role in the genetic differentiation.

The PCA (Figure 3) shows that the separation between the Staha formal system group and the on-farm sampled seed lots of Staha (*Staha local 1, Staha local 2* and *Staha recycled*) varies between almost complete overlap to no overlap across the two first components. The intermediate clustering of the simulated hybrid population between the formal system group and the local variety group shows the pattern of clustering expected if the two seed lots of local varieties included here had been representative for all potential sources of introgression. This is clearly not the case. However, the observed clustering pattern is compatible with the hypothesis that the genetic make-up of the recycled seed lot is due to gene flow from unrelated populations not sampled in this study. On the other hand, we also find support for the hypothesis that directional selection could have contributed to the distinctiveness of the recycled seed lots. We identify consistent and substantial allele frequency change across seed system stages for three out of 126 SNPs tested (<3%). In a large scale study of maize evolution, including more than 40 K SNPs genotyped in 400 individuals representing 80 years of modern breeding in North America, van Heerwaarden et al. (2012) [45] used the same Bayesian approach and identified a large number of markers under directional selection out of which many were located in genes of agronomic importance. In the survey part of this study, yield and drought tolerance are the most important characteristics reported, both as reason for growing the current varieties and as criteria for seed selection. The results suggest that drought tolerance is a trait under selection and that farmer management potentially leads to beneficial creolization in form of local adaptations. While two of the three loci identified here as being under directional selection in the local seed system are located in putative genes, their functions are unknown and we cannot conclude about their agronomic importance. Establishing links between patterns of genetic diversity and agronomic traits is found to be difficult both in studies of landraces and in studies of association populations. In a village level study of maize landrace management in Oaxaca, Mexico, it was demonstrated that even if the genetic differentiation was very low, the phenotypic differentiation was high for agronomically important quantitative traits [56]. Several recent studies of the Nested Association Mapping population [57] have shown that important agronomic traits are controlled by a large number of quantitative loci with small effects, and strong signatures of selection have proven to be elusive [58,59]. But while disentangling the effect of farmer management on local adaptation is difficult, our results suggest that both hybridization and selection contribute to the differentiation of OPVs as they enter the informal seed system.

## Conclusion

While in developed countries 98-100% of the maize area is planted with single-cross hybrids, most maize area in SSA is planted with local varieties, farm saved seeds of improved varieties or hybrids between the two [11]. The low adoption of improved seeds is often seen as a constraint to achieve increased maize production in the region and it is often used as a rationale for strengthening the formal seed system by measures like reforming seed registration and certification procedures and encouraging private sector involvement in the seed sector [11,13,17]. Other authors take the perspective that since the informal seed system is more important in supplying seeds than the formal system, efforts to enhance seed and food security should build upon current local seed systems and promote integration of formal and informal elements [19,60] with the ultimate goal of creating resilient and evolvable seed systems [23]. Smale et al. (2013) [9] warn against silver bullet solutions in the new initiatives to develop varieties and strengthen seed systems in Africa and stress the importance of learning from the long history of maize R&D programs on the continent. One such lesson is the importance of not only developing and promoting hybrid varieties, but also OPVs. In the Drought Tolerant Maize for Africa (DTMA) project managed by CIMMYT and IITA, 57 of the 105 varieties released in 13 countries since 2007 are OPVs [13]. While hybrids typically have higher achievable yield under recommended crop management, the OPVs are often more suitable in the socio-economic context of SSA’s smallholder agriculture. One of the reasons why OPVs play a prominent role in the current efforts is the fact that they can be farm-saved and recycled without a large yield penalty [21,61]. This case study provides molecular evidence of gene flow between improved varieties and local varieties of maize. This issue is often framed as a conservation concern, especially in the case of introduction of transgenic varieties [62,63]. There has been less focus on the potential role of introduced genetic material as a genetic resource in farmers’ seed management. In this paper we focus on implications for local seed systems. We show that OPVs bred for the drought susceptible semi-arid zone in Tanzania have entered the local seed system not only through adoption from formal seed channels, but also through gene flow and hybridization. We find molecular evidence of directional selection and survey evidence of farmer selection for drought tolerance. On this basis we conclude that access to genetic resources in the form of drought tolerant OPVs in combination with farmer seed selection is likely to enhance seed system security and farmers’ adaptive capacity in the face of climate change.

## Competing interests

The authors declare that they have no competing interests.

## Authors’ contributions

OTW, AKB and PRB designed the study and OTW drafted the manuscript. OTW and KHR analyzed the data. All authors contributed to and approved the final manuscript.

## Supplementary Material

Additional file 1: Table S1 SNPs used in this study. Click here for file

Additional file 2: Table S2 Information about maize varieties cultivated in the study area. Click here for file

Additional file 3: Table S3 Pairwise genetic differentiation (F_ST_) between seed lots. Click here for file
