# Supplementary material for: Modern maize varieties going local in the semi-arid zone in Tanzania
Source: BMC Evol Biol. 2014 Jan 2;14:1. doi: 10.1186/1471-2148-14-1 (PMC3890540; doi:10.1186/1471-2148-14-1)
Supplement: Additional file 2: Table S2 — Information about maize varieties cultivated in the study area. [file 1471-2148-14-1-S2.pdf]

Table S2. Details on the maize varieties grown in Mangae. Sources of information: Survey in Mangae July 2010 and National variety list 1950-2008 in (Ngwediagi et al., 2009).

**Variety**	**Year released and institution**	**Information**
Katumani	1950s by KARI Kenya	White Flint<br>Hybrid<br>Low rainfall areas<br>Suitable <1500 masl<br>Achievable yield: 3.0-3.5 tonns/ha
Kito	1983	Open pollinated variety (OPV)<br>White Flint<br>Early maturity: 90 days<br>Drought avoiding<br>Low rainfall areas
Staha	1983 by ARI Ilonga	OPV<br>White Flint-Dent<br>Based on cross between Ilonga composite (local breeding population) (50%), Tuxpeño (CIMMYT population) (45%) and small proportion (5%) of Katumbili<br>Large ears<br>Late maturity: 120 days<br>Drought tolerant<br>Good standability, not very tall<br>Tolerant to maize streak virus (MSV)<br>Suitable <900 maslAchievable yield: 4.0 -5.0 tonns/ha
TMV 1	1987 by ARI Ilonga	OPV<br>TMV 1: Tanzania Maize Variety 1<br>White Flint<br>Based on CIMMYT/IITA breeding population 11 (includes East African material and “La revolution” from Madagascar)<br>Intermediary maturity: 110 days<br>Resistant to MSV and rust<br>Suitable <1500 masl<br>Achievable yield: 4.5 tonns/ha
TAN250	Tanseed International	TAN 250 is based on CIMMYT’s ZM 401 selected for tolerance to drought and low soil fertility<br>Early maturity variety<br>Excellent resistance to MSV and Grey leaf spot, good resistance to Turcium leaf blight,<br>Cob rot and Common rust Suitable at low to medium altitude<br>Achievable yield: 3-5 tonns/ha
