# Supplementary material for: Modern maize varieties going local in the semi-arid zone in Tanzania
Source: BMC Evol Biol. 2014 Jan 2;14:1. doi: 10.1186/1471-2148-14-1 (PMC3890540; doi:10.1186/1471-2148-14-1)
Supplement: Additional file 3: Table S3 — Pairwise genetic differentiation (FST) between seed lots. [file 1471-2148-14-1-S3.pdf]

Table S3. Pairwise genetic differentiation between seed lots. Above diagonal: F_{ST} for comparison of the 12 sampled populations. Below diagonal: Significance level.

	1	2	3	4	5	6	7	8	9	10	11	12
Staha breeder's seed (1)		0.09167	0.01135	0.04792	0.08514	0.09249	0.13823	0.21596	0.14367	0.21758	0.08094	0.23902
Staha basic seed (2)	**		0.09901	0.15404	0.15629	0.17059	0.22064	0.32257	0.23049	0.30926	0.19453	0.35390
Staha commercial (3)	NS	**		0.06361	0.06316	0.11938	0.15275	0.21523	0.12308	0.23853	0.09576	0.27232
Staha local 1 (4)	**	**	**		0.12382	0.13746	0.13188	0.20784	0.17493	0.22251	0.09162	0.29642
Staha local 2 (5)	*	**	**	**		0.18467	0.18243	0.26865	0.22625	0.27931	0.14423	0.32304
Staha recycled (6)	**	**	**	**	**		0.19960	0.25750	0.20664	0.24842	0.12566	0.28529
TMV1 breeder's (7)	**	**	**	**	**	**		0.07903	0.03539	0.11639	0.15011	0.27140
TMV1 basic (8)	**	**	**	**	**	**	**		0.11742	0.17342	0.23597	0.37968
TMV1 commercial (9)	**	**	**	*	**	**	NS	*		0.12707	0.16039	0.36942
TMV1 local (10)	**	**	**	**	**	**	**	**	**		0.19960	0.36143
Local variety 1 (11)	**	**	**	**	**	**	**	**	**	**		0.26596
Local variety 2 (12)	**	**	**	**	**	**	**	**	**	**	**	

*Significant for p<0,05, **Significant for p<0,01, NS not significant
